# The Pew System

**Published:** 1863-07

**Authors:** 


### THE PEW SYSTEM.

STATISTICIANS have said that more persons kill themselves in summer-time than during the remainder of the year. Suicide is nearly always the result of mental suffering, of remorse, of worldly care, of disappointment, of misplaced confidence, of an abiding impression of forsakenness. The best balm for suffering hearts like these is found in the comforting and soothing influences of Christian doctrine; and these are to be found by the stranger, the troubled, and the outcast, in the house of God on the Sabbath-day, and that, too, without the necessity of exposing the secrets of their hearts to others, for these are fully known to Him who can be "touched with the feeling of our infirmities," and hence knows how to sympathize with his discouraged and suffering children. Multitudes more of such are found in cities and large towns, than in the village and the country church; and yet common custom has brought it about that a large number of the houses of public worship in cities are closed in the summer. This ought not to be. There can be no doubt that the regular incumbents severely need two or three months of recreation in the country, of entire release from pastoral duty at home, of rest for the mind, and renovation for the body. But a great good could be accomplished in various ways, and no time lost, if there were to be an interchange of labor between city and country pastors; for the three summer months, each could preach without special study; each could enjoy a change of air, and food, and associations, and exercises; while the members of exchanging churches would have a variety of *spiritual* aliment not altogether without its good uses. It would answer a great good purpose also to have it universally known that during the hot months, all houses of religious worship should be absolutely free to all comers; this arrangement would not only be a return to apostolic times, when "the poor have the Gospel preached to them," but it would aid in filling up depleted congregations, and thus enable ministers to officiate with greater ease, comfort, and encouragement; for it is well known that the fuller any house is, not only is it easier to preach, but there is an influence in large numbers, in crowded houses, which predisposes the mind to the more eager and profitable reception of the truth which may be presented.

It must not be said that the public are welcome to seats in our churches; they are not welcome to any first-class church in New-York City. Doubtless, the public are welcome theoretically; and, in fact, pains are taken to advertise in the public papers very extensively, that religious services will be held at such a place and such an hour, and "respectfully invited to attend," is the stereotype phrase. No doubt the minister's heart would be delighted to have the house filled from floor to dome; no doubt every member of the society, parish, or congregation would be highly pleased to see the whole house full—provided, yes, provided, he had his own pew all to himself and his own family. Such persons would be even glad to give up their own seats to any eminent citizen or distinguished name. But as for being pleased to find his pew occupied by John Smith and all the little Smiths, and the great big red-faced Mrs. Smith, that is out of the question, even though this same John Smith and family were "known of all" to be a very fine family and without reproach. But now for the proof, for doctors always like to clinch their statements with "cases" in point, which have come under their own personal observation.

Now we give the reader notice beforehand, that we are going to be personal and specific; a kind of spice necessary to make an article for a July day at all readable. We must, however, state first and foremost, in self-defense, that we seldom go to any other church than our own; and the worse the weather, the more need we feel it to be a kind of holding up of the hands of our worthy minister; and there is more in this statement than would seem to be on the surface; for to attend church twice a day on the Sabbath, requires of us a six-mile walk along Fifth Avenue; which is as great a test of faith under the broiling sun of July as in the zero of mid-winter. If we do not go to our own church, it is because there is "no meeting" there, or we are "professionally engaged," or otherwise called away. When our house is closed, we have sometimes, under a strong outside pressure, such as a public announcement, or flaming advertisement, or to gratify some visitor to our city, gone to other places, but not with very agreeable results always.

The only prominent church in New-York in which we have seen things done in the right way is at "Brother Chapin's," that is, Henry Ward Beecher's "Brother," not ours! As he believes that all are to be saved, sooner or later, (it will be very late as to some fellows we know,) he and his leading men invite all to come, and welcome all who enter their doors, by having some half a dozen of their leading men to stand at the entrance of the aisles for the purpose of showing strangers to convenient seats without waiting a moment; this is Christian politeness; this is a true civilization.

To begin at our own church, (an editor takes the privilege of seeing and hearing all he can, and also of making any practical use of it he chooses, by making it fit in wherever suits him best,) we once heard a rich Broadway merchant blowing up our worthy sexton, "before folks," for having shown a stranger into his pew, which was only occupied besides by himself and handsome young wife. On another occasion, a poor old "brother" who used to sit in the gallery came down to the communion by invitation, but got into the "wrong pew" that time, for it belonged to a "sister" in the church, and the way she did blaze away for his presuming to occupy her seat without being invited, would have astonished the pastor who had delivered to her the "elements" not half an hour before! It turned out that the poor old man never came to church afterward, for he sickened and died the same summer, and, no doubt, went up higher than that sister will ever get.

We have before named our adventure with a gifted lady on our arm, who particularly wanted to hear the celebrated incumbent of the All Souls' or All Saints' striped building on Fourth Avenue. There were not fifty people in the pews, but there were more at the door-way, waiting to be shown to seats; for the public had been "invited to attend" through the Saturday papers. The sexton must have thought there was an endless job before him, and in despair of getting through with the increasing crowd, he made a tremendous sweep of his arm, exclaiming, at the same time: "There's plenty of room in the gallery." We have several times been to the Brick Church on Murray Hill, Fifth Avenue, and to the best of our present recollection, never have been invited to a seat; no doubt we would have been, had we waited long enough. But when there are even ladies standing, at the end of the "long prayer," it is time to accommodate yourself, which we have uniformly done by going up into the gallery and sitting on the steps, whence we have seen persons still waiting for a seat at the commencement of the sermon; this was, however, on the occasion of a splendid doctrinal discourse by that eminently able and gifted divine, the Rev. N. L. Rice, D.D., whose power of lucid explanation of scripture doctrine is not equaled by any clergyman we have ever listened to at home or abroad. A one-horse sexton is not enough for our large churches; there ought to be two men, (or women ushers, as we have seen in London,) at the head of each aisle, until the moment of taking the text, when the outer doors should be closed. But worse than any thing yet happened to us last summer. We had for a long time wanted to hear that great and good man, the Rev. Dr. Williams, (Baptist;) but his church was "closed for the summer." On our way home, we chanced to pass the "Mercer-street Church," and recollecting that the secular papers of the day before had announced that a distinguished scholar and divine would officiate, whom we had long wanted to hear, we stepped in; at least half the pews had not a single occupant, and as we saw no sexton about, we concluded that the seats were "free indeed," and took one near the door, on the left-hand side of the central aisle; such seats being usually appropriated to strangers, loungers and "niggers;" and being the only occupant, with our little boy of eight years, we forthwith became absorbed in the minister, in trying to find an answer to the question: "What man is that?" Not for the words that came out of his mouth, but for the manner. Meanwhile we had most completely forgotten the fact of the public announcement; for we soon found ourself almost audibly exclaiming: "This certainly can't be the pastor of this church." "No city congregation could by any possibility have elected such a concentration of affectedness as that." Then the mind got mixed up entirely; for it ran off into a balancing of the question of deformity, as if we were looking at Mr. Williams, who has some bodily defect; but his mind how grand and lofty! The speaker was all crouched down, as if he were going to make a hoop out of himself. He was reading a portion of Scripture. His nose almost touched the book; one shoulder seemed to be about a foot long and pointed with a very obtuse angle down into the cellar; the other about two inches long, looked sky high; then we transmogrified ourself into an inquisitive old-time Yankee—that is to say, a pharisee; and wanted to know if "this man were born so," or if not, what kind of an accident could have induced such a deformity, or what sin had he committed, that such a thing should have befallen him. And then the manner of his reading; it was in that soft, smooth, measured, oily manner and tone, which is apt to be assumed by those who are deliberately attempting to deceive, or to charm. We make no charges; and only wish to state what was passing in the mind; but before we could come to any conclusion that was at all satisfactory, we were tapped on the shoulder, and looking round, there was the sexton, who in a very civil manner asked if a seat nearer the pulpit would not be more pleasant? The reader will know how quick thoughts can fly sometimes, and how soon the mind jumps to conclusions. We felt pleased at the consideration of the sexton, and flattered also at his attention; thinking, in fact—shall it be confessed?—that he saw something about us which led him to believe we were not a common individual. And as he was the first person whose optics were sharp enough

"To see what was not to be seen,"

we were about setting him down in the book of our estimation as a very smart man; and expressed, with a respectful declination, our obligation for his consideration. "But," said the worthy man, "the owner wants to occupy the pew himself." Now we didn't get mad as fire. That would neither have been wise nor profitable nor becoming. In truth, it would have been "infra dig." The thought ran through the mind a hundred times quicker than can be expressed. "It's all right! very natural that a man should want his own pew;" and half feeling that we ought not to have entered it, we whispered to the "owner" on leaving it, that we would go nearer the pulpit, where we could hear better. This was said for two reasons: first, that his feelings should not be hurt by our leaving the pew altogether; and second, we were willing enough to get nearer; as we enjoy religious services better, the nearer we can get to the pulpit. We heard an excellent, able, pertinent and instructive discourse; the minister stretched himself out "as straight as a shingle;" his voice was distinct and manly; his shoulders square and even and well balanced, and we went home, glad to have gone where we did. But what do you think, reader? when we got home, we found old "Clooty" there, and he set us thinking over all that had passed, and would you believe it? we feel some inklings of irritation every time we think of it to this day. There were at least a dozen pews near without a single occupant; and the "owner" ought to have felt free enough to have entered his own pew, or occupy any of the vacant ones. How long he may have stood waiting for us to see him, we can't say; he might have stood to the end of the discourse, for, as we stated, we were in serious quandaries; in an investigating turn of mind; were, in short, in the pursuit of knowledge under difficulties. We have not written this article as an abuse of the pew system, for it may be necessary under all the circumstances of the times. But it does seem more in accordance with the essence of the Christian religion, which is brotherly kindness and charity, that it should be offered to all, to the poor and the distressed, the stranger and the helpless, with the same "freedom" as it was given, which was without money and without price. The great pioneer Church, the Methodist, has flourished under the free-pew system, far beyond that of any other evangelical denomination; the Society of Friends have free seats; with them the beggar has as absolute right to a place in their meeting-house as any prince or potentate. At all events, we close with two suggestions: first, let city churches be literally free to all during the three summer months; second, hold out no false light of inviting the public to attend through the public papers when they must stand waiting a quarter of an hour, and when invited to a seat, may be invited also to get up and march and counter-march two or three times during the discourse, to let in the "owner's" family.

